# Hydrogen Peroxide Produced by Oral Streptococci Induces Macrophage Cell Death

**DOI:** 10.1371/journal.pone.0062563

**Published:** 2013-05-03

**Authors:** Nobuo Okahashi, Masanobu Nakata, Tomoko Sumitomo, Yutaka Terao, Shigetada Kawabata

**Affiliations:** 1 Department of Oral Frontier Biology, Osaka University Graduate School of Dentistry, Suita-Osaka, Japan; 2 Department of Oral and Molecular Microbiology, Osaka University Graduate School of Dentistry, Suita-Osaka, Japan; 3 Division of Microbiology and Infectious Diseases, Niigata University Graduate School of Medical and Dental Sciences, Niigata, Japan; University of Malaya, Malaysia

## Abstract

Hydrogen peroxide (H_2_O_2_) produced by members of the mitis group of oral streptococci plays important roles in microbial communities such as oral biofilms. Although the cytotoxicity of H_2_O_2_ has been widely recognized, the effects of H_2_O_2_ produced by oral streptococci on host defense systems remain unknown. In the present study, we investigated the effect of H_2_O_2_ produced by *Streptococcus oralis* on human macrophage cell death. Infection by *S. oralis* was found to stimulate cell death of a THP-1 human macrophage cell line at multiplicities of infection greater than 100. Catalase, an enzyme that catalyzes the decomposition of H_2_O_2_, inhibited the cytotoxic effect of *S. oralis*. *S. oralis* deletion mutants lacking the *spxB* gene, which encodes pyruvate oxidase, and are therefore deficient in H_2_O_2_ production, showed reduced cytotoxicity toward THP-1 macrophages. Furthermore, H_2_O_2_ alone was capable of inducing cell death. The cytotoxic effect seemed to be independent of inflammatory responses, because H_2_O_2_ was not a potent stimulator of tumor necrosis factor-α production in macrophages. These results indicate that streptococcal H_2_O_2_ plays a role as a cytotoxin, and is implicated in the cell death of infected human macrophages.

## Introduction

Members of the oral mitis group of streptococci are causative agents of oral biofilm, dental plaque, and infective endocarditis [1], [2], [3], [4]. *Streptococcus oralis*, *Streptococcus sanguinis*, and *Streptococcus gordonii* are members of the mitis group of oral streptococci and primary colonizers of the human oral cavity [1], [2], [3], [4]. These oral streptococcal species are known to produce hydrogen peroxide (H_2_O_2_) [1], [2], [5], [6], with the H_2_O_2_ produced playing important roles in microbial communities such as oral biofilms [6], [7]. *S. sanguinis* and *S. gordonii* have been reported to produce H_2_O_2_ at concentrations sufficient to reduce the growth of many oral bacteria, including the cariogenic *Streptococcus mutans* [7]. H_2_O_2_ also stimulates the release of bacterial DNA, which appears to support oral biofilm formation and facilitate gene exchange amongst bacteria [8].

The oral mitis group of streptococci is known to cause a variety of infectious complications, including bacteremia and infective endocarditis [9], [10], [11], [12]. Studies by the United Kingdom’s Health Protection Agency have shown that the rate of bacteremia caused by the mitis group of streptococci is comparable to that of group A or group B streptococci [13]. Furthermore, epidemiological studies have shown the presence of these streptococcal species in heart valve and atheromatous plaque clinical specimens [14], [15], [16].

Macrophages and monocytes are major contributors to host immune responses against bacterial infections. Although oral streptococcal species are known to cause bloodstream infections and infectious endocarditis, their pathogenicity toward macrophages is not well understood. We previously found that *S. sanguinis* induces foam cell formation and macrophage cell death, and that its cytotoxicity is likely to be associated with reactive oxygen species [17]. Further study suggested that the macrophage cell death is related to H_2_O_2_ production by the streptococcal species. Although the cytotoxicity of H_2_O_2_ has been widely recognized, the effects of H_2_O_2_ produced by oral streptococci on host defense systems remain unknown.

In the present study, we investigated whether H_2_O_2_ produced by the oral mitis group of streptococci is implicated in infected human macrophage cell death.

## Materials and Methods

### Bacterial strains and culture conditions

*S. oralis* ATCC35037, a type strain originally isolated from human mouth [18], was obtained from the Japan Collection of Microorganisms at the RIKEN Bioresource Center (Tsukuba, Japan). *S. mutans* MT8148 and *Streptococcus salivarius* HHT were selected from the stock culture collection in the Department of Oral and Molecular Microbiology, Osaka University Graduate School of Dentistry. *S. sanguinis* SK36 was provided by Dr. M. Killian (Aarhus University, Denmark). These bacteria were cultured in Brain Heart Infusion (BHI) broth (Becton Dickinson, Sparks, MD, USA). *Escherichia coli* strain XL10-gold (Stratagene, La Jolla, CA, USA) was grown in Luria-Bertani broth.

### Cell culture

The human monocyte cell line THP-1 cells were purchased from RIKEN Bioresource Center and cultured in RPMI1640 medium (Invitrogen, Carlsbad, CA, USA) supplemented with 5% fetal bovine serum (FBS) (Invitrogen) (5% FBS RPMI1640), penicillin (100 U/ml), and streptomycin (100 µg/ml) at 37°C in a 5% CO_2_ atmosphere. Differentiated THP-1 macrophages were prepared by treating THP-1 cells with 100 nM phorbol myristate acetate (PMA) (Sigma Aldrich, St. Louis, MO, USA) for 2 days.

### Cell death of macrophages

Differentiated THP-1 macrophages (2×10^5^ cells in 5% FBS RPMI1640) were infected with viable streptococcal strains at a multiplicity of infection (MOI) of 50, 100, or 200, in the absence of antibiotics, for 2 h. Cells were washed with phosphate buffered saline (PBS, pH 7.2) to remove extracellular non-adherent bacteria, and cultured for 18 h in fresh medium containing antibiotics. Macrophages were then stained with 0.2% trypan blue (Sigma Aldrich) in PBS. After incubation at room temperature for 5 min, the numbers of viable and dead cells were counted using a microscope (Nikon TMS-F, Nikon, Tokyo, Japan).

Cell death induced by H_2_O_2_ was determined similarly. Differentiated THP-1 macrophages were cultured in the presence of 1, 5, or 10 mM H_2_O_2_ (Nacalai Tesque, Kyoto, Japan) for 18 h, and viability was determined by trypan blue staining.

### Effect of catalase on cell viability

Prior to infection, 10 or 100 U/ml of catalase (Sigma-Aldrich) was added to the cultures of differentiated THP-1 macrophages, and cells were then infected with viable *S. oralis* strains (MOI; 50, 100, or 200) for 2 h. Cells were washed with PBS, and cultured in fresh medium containing catalase and antibiotics for 18 h. Viability was determined as described above.

### Construction of *spxB*-deficient mutant

The DNA sequence for the pyruvate oxidase gene (SpxB) of *S. oralis* ATCC35037 (*SMSK23_0092*) was obtained from Gene Bank (accession number NZ_AEDW01000001). The *spxB* locus was deleted using a temperature-sensitive suicide vector pSET4s [19], as reported previously [20], [21], [22]. For construction of the *spxB* deletion mutant, spxKO-F1 and spxKO-R1 primers (Table S1) were utilized for PCR amplification of the upstream flanking sequence of the *spxB* gene. The downstream flanking sequence of the *spxB* gene was amplified using primers spxKO-F2 and spxKO-R2 (Table S1). By using the 2 generated PCR products containing complementary ends, overlap PCR was performed with the primers spxKO-F1 and spxKO-R2. The overlap PCR product was digested with *Eco*RI and *Bam*HI and cloned into the pSET4s vector via *Eco*RI/*Bam*HI sites. The resultant plasmid pSET4s-*spxB*KO was transfected into *S. oralis* ATCC35037 by electroporation. Transformants were grown at 28°C and selected on BHI agar plates containing spectinomycin (100 µg/ml). Single-crossover mutants were obtained by culturing the cells on agar plates with spectinomycin at 37°C, and double-crossover mutants were generated by repeated passaging on agar plates with no antibiotic at 28°C. Finally, spectinomycin-sensitive colonies were tested for deletion of the *spxB* gene by PCR using primers spx-inside-F/-R and spx-outside-F/-R (Table S1). The *S. oralis* glucosyltransferase (*gtfR*) gene was used as a positive control (Table S1) [23]. During the course of the double-crossover, both the *spxB*-deletion mutant (*spxB* KO) and the revertant mutant (*spxB* Rev), which possesses the wild-type allele, were generated from the same ancestor. To rule out the effects of secondary mutations that may have arisen during mutagenesis, a revertant strain was used as a control. Original strain of *S. oralis* ATCC35037 was used as a wild type (WT) strain.

### Hydrogen peroxide measurement

H_2_O_2_ in *S. oralis* culture media was quantitatively determined using a hydrogen peroxide colorimetric detection kit (ENZO Life Science, Plymouth Meeting, PA, USA). *S. oralis* WT, *spxB* KO, and *spxB* Rev strains were cultured in BHI broth or RPMI1640 medium supplemented with 5% FBS for 18 h. Culture supernatants were diluted 50-fold in PBS, and H_2_O_2_ concentrations were then determined according to the manufacturer’s instructions. Our preliminary experiments suggested that, without sufficient dilution, both BHI broth and RPMI1640 medium interfered with the colorimetric reaction of the kit.

### Fluorescence microscopy

Differentiated THP-1 cells were cultured on gelatin-coated chamber slides (Asahi Glass, Tokyo, Japan). The macrophages were exposed to *S. oralis* WT, *spxB* KO, and *spxB* Rev strains at an MOI of 200 for 2 h, washed with PBS to remove extracellular bacteria, and cultured for an additional 18 h. The cells were washed with PBS, and then stained by Live/Dead Staining Kit (PromoCell, Heiderberg, Germany). Stained cells were analyzed using an LSM 510 confocal laser microscope (Carl Zeiss, Oberkochen, Germany). Ethidium homodimer III (EthD-III) (red fluorescence) stained the nuclear DNA of dead THP-1 cells, while calcein AM (green fluorescence) stained live cells. THP-1 cells treated with H_2_O_2_ were stained and observed in a similar manner.

### TNF-α assay

Differentiated THP-1 macrophages were infected with viable *S. oralis* WT, *spxB* KO, and *spxB* Rev strains (MOI; 50, 100 or 200) in the absence of antibiotics for 2 h. Cells were washed with PBS to remove extracellular bacteria, and cultured in fresh medium containing antibiotics for an additional 18 h. Cells were also subject to different concentrations of H_2_O_2_ (1, 5, and 10 mM). The amount of tumor necrosis factor-α (TNF-α) in culture supernatants was measured using ELISA kits (Thermo Scientific, Waltham, MA, USA) according to the manufacturer’s instructions.

### Statistical analysis

Statistical analyses were performed using QuickCalcs software (GraphPad Software, La Jolla, CA, USA). Experimental data are expressed as the mean ± SD of triplicate samples. Statistical differences were examined using independent Student’s *t*-test, with *p*<0.05 considered to indicate statistical significance.

## Results

### *S. oralis* induces cell death of THP-1 macrophages

**Figure 1:**
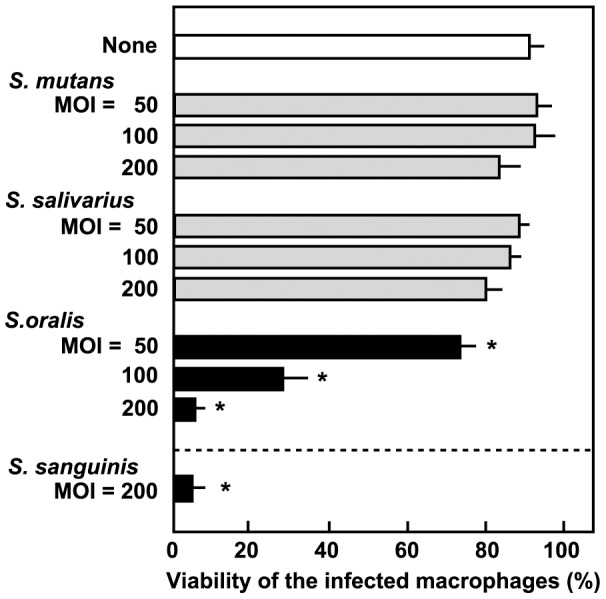
THP-1 macrophage cell death induced by oral streptococci. Differentiated THP-1 macrophages were infected with viable *S. mutans* MT8148, *S. salivarius* HHT, and *S. oralis* ATCC35037 for 2 h; washed with PBS to remove non-adherent extracellular bacteria; and cultured in fresh medium containing antibiotics for 18 h. As a control, macrophages were also infected with *S. sanguinis* SK36 [17]. Macrophage viability was determined by a trypan blue dye exclusion method. Data are shown as the mean ± SD of triplicate samples. *p<0.05 as compared with untreated control (None).

We previously reported that infection with *S. sanguinis* induces THP-1 macrophage cell death, with reactive oxygen species apparently contributing to this process. [17]. In the present study, we first examined whether other oral streptococcal species also induce macrophage cell death. Differentiated THP-1 macrophages were exposed to viable oral streptococcal strains, *S. mutans* MT8148, *S. salivarius* HHT, and *S. oralis* ATCC35037. Macrophages were then stained with trypan blue to determine their viability (Figure 1). At an MOI of more than 100, viable *S. oralis* induced cell death of macrophages at a level comparable to *S. sanguinis* [17]. Exposure to *S. mutans* or *S. salivarius* showed little effects on the viability of the macrophages even at MOIs of 200. During infection at an MOI of over 500, all tested streptococci steadily induced cell death (data not shown). This was likely due to acidification of culture medium and/or accumulation of cytotoxic products such as formic and acetic acids [1], [2], [24].

**Figure 2:**
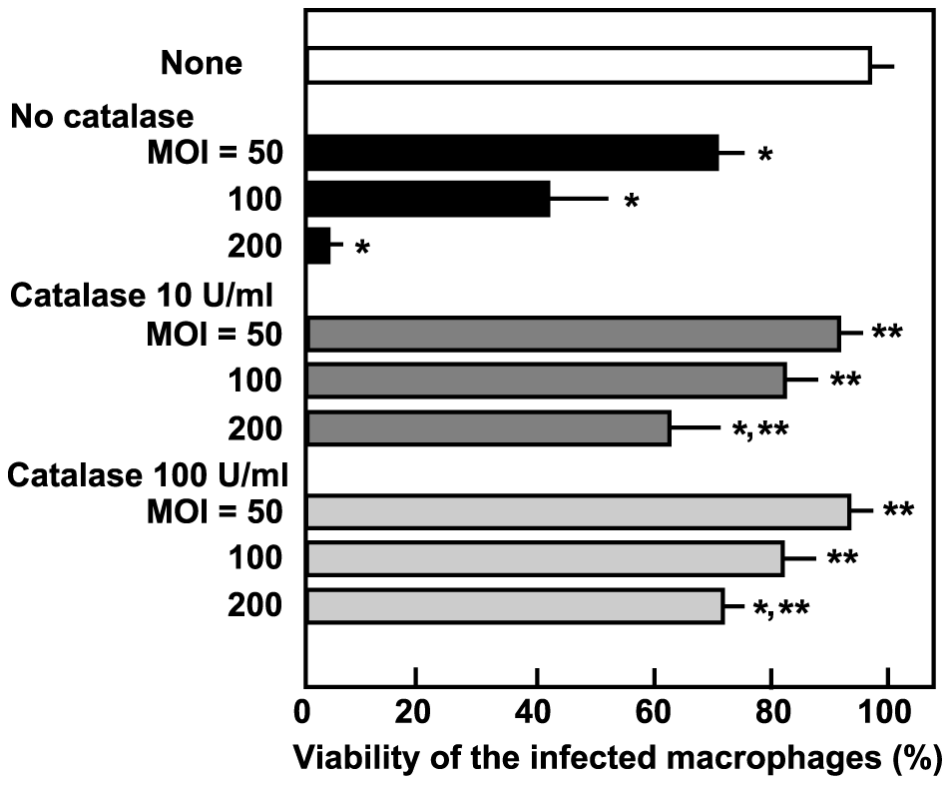
Effect of catalase on macrophage cell death. Prior to infection, either 10 or 100 U/ml of catalase was added to cultures of differentiated THP-1 macrophages, and cells were then infected with viable *S. oralis* ATCC35037 (MOI: 50, 100, or 200) for 2 h. Cells were washed with PBS and cultured in fresh medium containing catalase and antibiotics for 18 h. Viability was determined by a trypan blue dye-exclusion method. Data are shown as the mean ± SD of triplicate samples. *p<0.05 as compared with untreated control (None). **p<0.05 as compared with the cells infected at the same MOI without catalase.

It is well known that *S. oralis* and *S. sanguinis* produce H_2_O_2_, whereas *S. mutans* and *S. salivarius* do not [1], [2]. Because reactive oxygen species were previously shown to contribute to cell death of macrophages [17], we investigated the effect of catalase, an H_2_O_2_-decomposing enzyme, on *S. oralis*-induced cell death. Exogenously added catalase was shown to reduce cell death in macrophages infected with *S. oralis* ATCC35037 (Figure 2), suggesting that H_2_O_2_ is involved in this process.

### Construction of *spxB* deficient mutant

**Figure 3:**
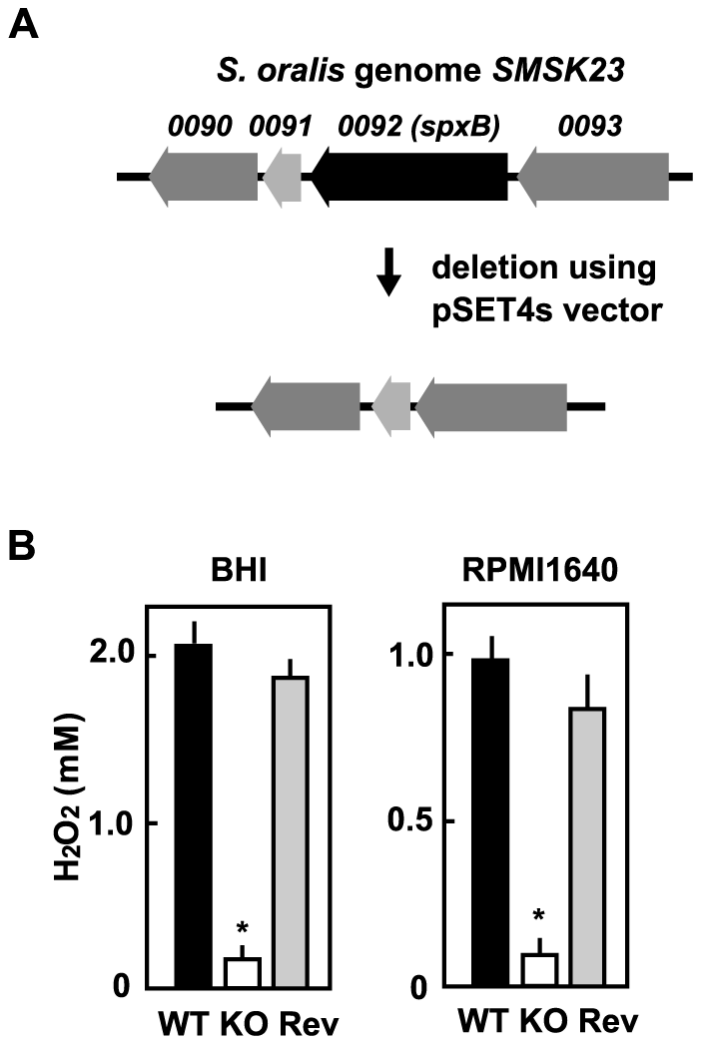
Construction of *S. oralis* spxB deletion mutant. (A) Black arrow indicates the gene encoding pyruvate oxidase (SMSK23_0092 spxB). A targeted deletion mutant lacking this region was constructed by allelic exchange using the temperature-sensitive shuttle vector pSET4s. (B) S. oralis ATCC35037 wild-type (WT), spxB-deletion mutant (KO), or reverse mutant (Rev) was cultured in BHI broth or 5% RPMI1640 medium at 37°C for 18 h in a 5% CO_2_ atmosphere. Concentrations of H_2_O_2_ in culture supernatants were quantitatively determined using a hydrogen peroxide colorimetric detection kit. Data are shown as the mean ± SD of triplicate samples. *p<0.05 as compared with concentration of wild-type strain.

Pyruvate oxidase has been reported as being essential for H_2_O_2_ production in the mitis group of streptococci [5], [6], [25]. Therefore, we constructed a deletion mutant of the pyruvate oxidase gene, *spxB*, via allelic exchange by using a temperature-sensitive shuttle vector (Figure 3A). Deletion of the *spxB* gene in the mutant was verified by PCR (data not shown). Decreased production of H_2_O_2_ by the deletion mutant (*spxB* KO) was confirmed both in BHI broth and RPMI1640 medium containing 5% FBS at 37°C in a 5% CO_2_ atmosphere (Figure 3B). The production of H_2_O_2_ by the *spxB* revertant mutant (*spxB* Rev) was similar to that of a wild type (WT) strain. The mutant strains grew at rates comparable to those of the WT strain (data not shown).

### Contribution of H_2_O_2_ produced by *S. oralis* to macrophage cell death

**Figure 4:**
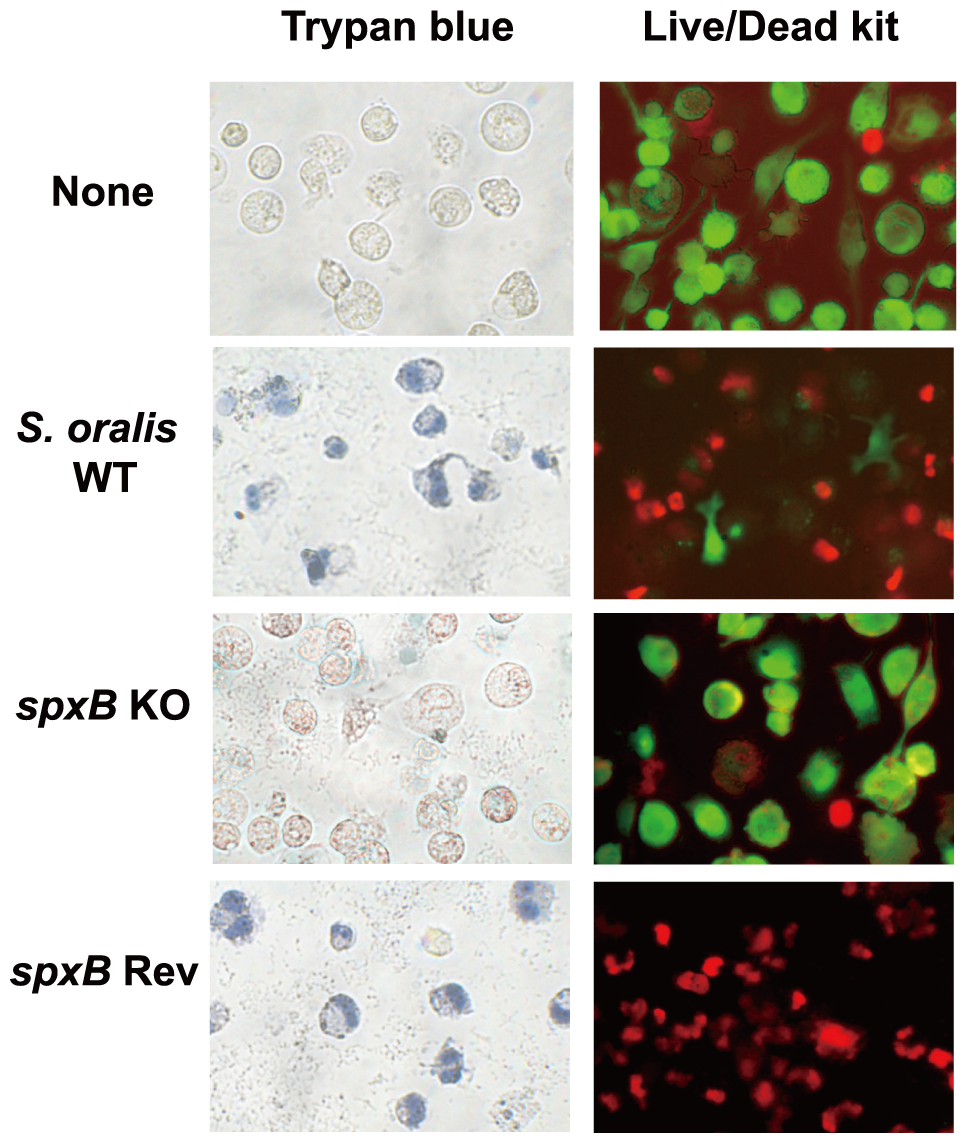
Microscopic images of macrophage cell death. THP-1 macrophages were infected with *S. oralis* wild-type strain (WT), mutant strain defective in H_2_O_2_ production (*spxB* KO), or reverse mutant strain (*spxB* Rev) for 2 h, washed with PBS, and cultured in fresh medium containing antibiotics for 18 h. Macrophages were stained with trypan blue and Live/Dead cell staining kit. EthD-III (red fluorescence) stained the nuclear DNA of dead THP-1 cells, while calcein AM (green fluorescence) stained live cells. Bar, 50 μm.

In order to evaluate the contribution of H_2_O_2_ produced by *S. oralis* to macrophage cell death, differentiated THP-1 cells were exposed to *S. oralis* WT strain, *spxB* KO mutant, and *spxB* Rev mutant. Macrophages were then stained with trypan blue to determine their viability (Figure 4, left). At an MOI of 200, macrophages infected with *S. oralis* WT and *spxB* Rev strains were found dead, whereas most of the *spxB* KO-infected cells were still viable. Live/Dead fluorescence staining also revealed reduced cell death of macrophages infected with *spxB* KO mutant (Figure 4, right).

**Figure 5:**
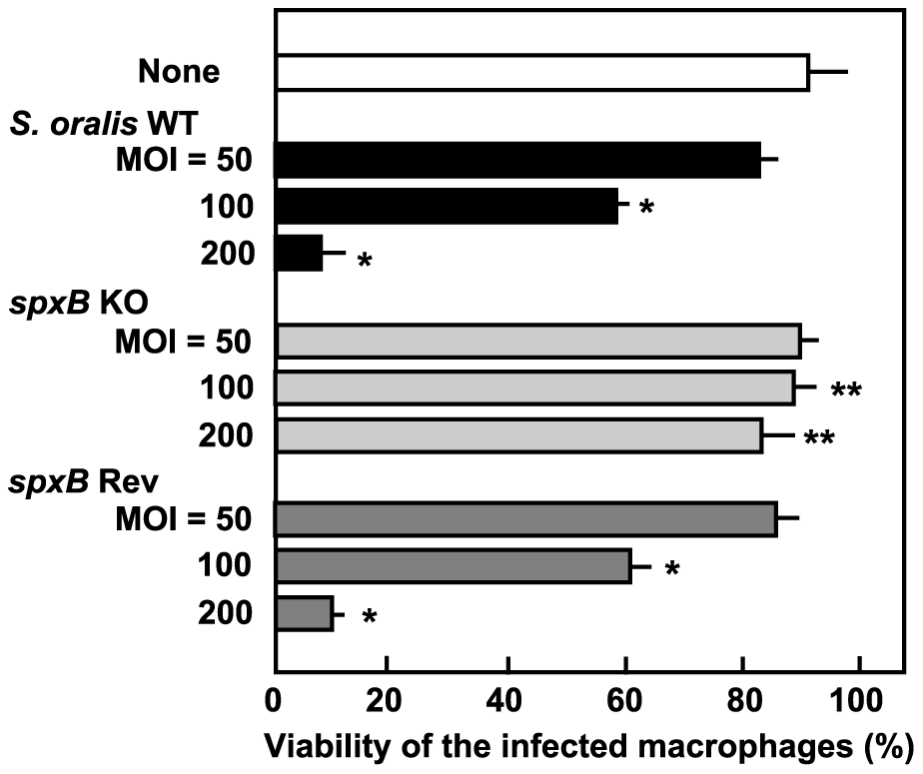
Deletion of *spxB* gene reduces *S. oralis* cytotoxicity. THP-1 macrophages were infected with *S. oralis* wild-type strain (WT), mutant strain defective in H_2_O_2_ production (*spxB* KO), or reverse mutant (*spxB* Rev) for 2 h, washed with PBS, and cultured in fresh medium containing antibiotics for 18 h. Macrophage viability was determined by a trypan blue dye exclusion method. Data are shown as the mean ± SD of triplicate samples. *p<0.05 as compared with untreated control (None). **p<0.05 as compared with the cells infected with WT at the same MOI.

*S. oralis* WT and *spxB* Rev strains induced THP-1 macrophage cell death in a dose-dependent manner (Figure 5). On the other hand, *spxB* KO mutants had a reduced cytotoxic effect, even at an MOI of 200, indicating that H_2_O_2_ produced by *S. oralis* contributes to the induction of macrophage cell death.

**Figure 6:**
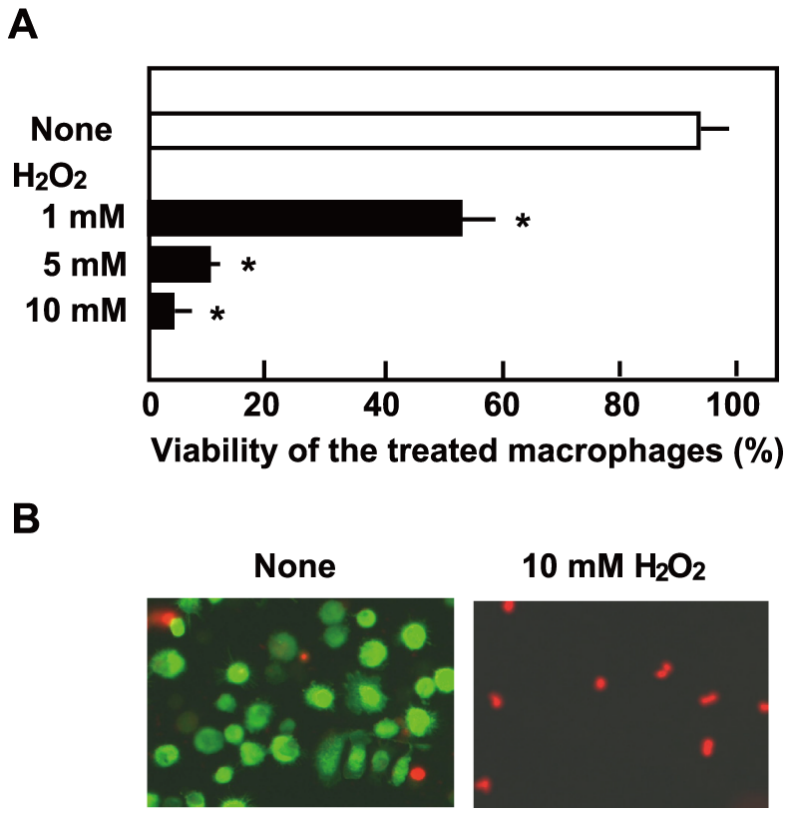
Cell death induced by H2O2. (A) Differentiated THP-1 macrophages were cultured in the presence of 1, 5, or 10 mM H_2_O_2_ for 18 h, and their viability was determined by the trypan blue staining. Data are shown as the mean ± SD of triplicate samples. *p<0.05. (B) Macrophages treated with 10 mM H_2_O_2_ were stained with Live/Dead cell staining kit. EthD-III (red fluorescence) stained the nuclear DNA of dead cells, while calcein AM (green fluorescence) stained live cells. Bar, 50 µm.

To confirm that H_2_O_2_ is, in itself, sufficient to induce cell death, THP-1 macrophages were incubated with H_2_O_2_ alone. As shown in Figure 6, the addition of H_2_O_2_ to THP-1 cell cultures induced cell death in a dose-dependent manner.

#### Effect of H_2_O_2_ on TNF-α production in THP-1 macrophages

**Figure 7:**
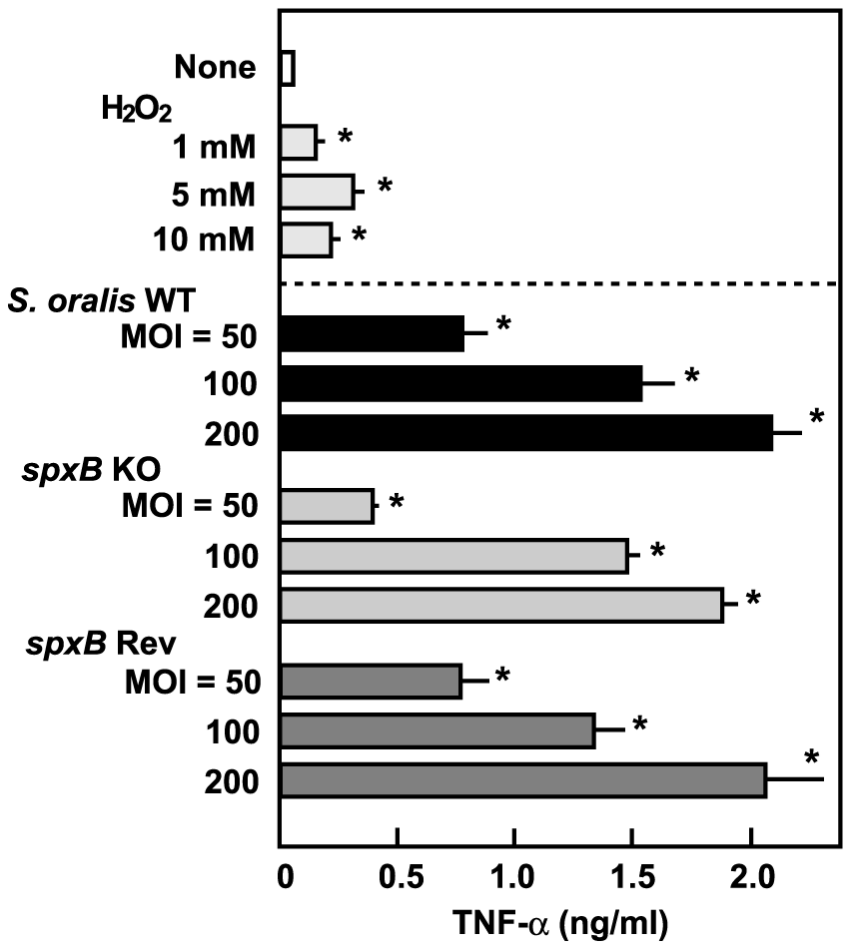
Induction of TNF-α by *S. oralis spxB* KO mutant. Differentiated THP-1 macrophages were infected with viable *S. oralis* strains for 2 h, and then washed and cultured for additional 18 h. Other cultures were stimulated by exposure to H_2_O_2_. The release of TNF-α was determined using an ELISA kit. Data are shown as the mean ± SD of triplicate samples. *p<0.05 as compared with untreated control (None).

It is widely recognized that microbial stimulation induces cytokine production in macrophages. Infection with viable *S. oralis* WT strain induced the production of an inflammatory cytokine, TNF-α (Figure 7). The amount of TNF-α in macrophage culture supernatants increased in a dose-dependent manner. No significant differences in cytokine production between macrophages infected with either WT or *spxB* Rev strains and those infected with *spxB* KO mutants were observed. Furthermore, H_2_O_2_ on its own had a limited stimulatory effect on TNF-α production (Figure 7). These results suggest that H_2_O_2_ is not essential to TNF-α production in *S. oralis*-infected macrophages.

## Discussion

In our previous study, we showed that *S. sanguinis*, a member of the oral mitis group of streptococci, induces macrophage cell death [17]. Since *S. sanguinis* have no established cytotoxins [1], [2], this finding was unexpected. Here, we confirmed that infection with viable *S. oralis*, another member of oral mitis group, also induced THP-1 macrophage cell death. The most important finding in this study was that streptococci-derived H_2_O_2_ exhibited cytotoxicity to macrophages.

The oral mitis group of streptococci can give rise to a variety of infectious complications, including bacteremia and infective endocarditis [10], [11], [12], [13]. These bacteria frequently enter the bloodstream following trauma to oral tissues, and then colonize to heart valve surfaces [2], [9], [10], [11]. The mitis group of oral streptococci is the most common cause of native valve endocarditis in humans, accounting for over 30% of cases [9], [10], [11]. The cytotoxicity and tissue-damaging effects of streptococcal H_2_O_2_ may be factors of bacterial pathogenicity. It is likely that the cytotoxic effect of H_2_O_2_ enables bacteria to escape from macrophage phagocytosis, and thus contribute to the onset of bacteremia and infectious endocarditis. Furthermore, dead macrophages are reportedly involved in atherosclerosis plaque development [26]. In infective endocarditis, oral streptococci in the bloodstream are entrapped in the platelet-fibrin matrix of cardiovascular tissue vegetations [12], [14]. In such infected lesions, H_2_O_2_ produced by the streptococci might damage host tissues and allow the bacteria to evade host defense mechanisms. Thus, streptococcal H_2_O_2_ should be considered as a cytotoxin, and H_2_O_2_-producing enzymes could be potent targets of the treatments of infections by mitis group of streptococci. Although the H_2_O_2_ generated by damaged mitochondria is known to induce cell death in various ways [27], our study using an *spxB* KO mutant strongly suggested that H_2_O_2_ of bacterial origin plays a major role in macrophage cell death.

Several investigations into *Streptococcus pneumoniae*, a pathogenic member of the mitis group of streptococci, have reported that bacterial H_2_O_2_ production is a factor of bacterial pathogenicity. H_2_O_2_ is suggested as contributing to pneumococcal lung and blood infections in experimental animals [25]. Another study showed that H_2_O_2_ produced by *S. pneumoniae* induces microglial and neuronal apoptosis *in vitro*, and infection with a pneumococcal *spxB* KO mutant reduces the severity of experimental pneumococcal meningitis [28]. Bioluminescent imaging in infected mice has shown that SpxB contributes to prolonged nasopharyngeal colonization of *S. pneumoniae* [29]. These studies also indicate that H_2_O_2_ plays a role as a bacterial cytotoxin. It is therefore conceivable that the H_2_O_2_ produced by oral streptococci contributes to their virulence. In fact, Stinson et al. [30] reported that the addition of catalase protected endothelial cells from cell death induced by *S. gordonii*, suggesting that the H_2_O_2_ produced by the bacteria may contribute to cell death.

The molecular mechanisms underlying streptococcal H_2_O_2_-mediated cell death are not well understood. H_2_O_2_ is widely employed as a general-purpose disinfectant. Cell membranes are permeable to H_2_O_2_, which causes toxicity via oxygen formation, lipid peroxidation, and damage to proteins and nucleic acids [31]. Our previous study using *S. sanguinis* [17] showed that this cell death is independent of caspase-1 activation. Braun et al. [28] have suggested that pneumococcal H_2_O_2_ induces apoptosis through release of apoptosis-inducing factor (AIF) from mitochondria in human microglia cells. However, their study showed that the cholesterol-dependent cytolysin, i.e., pneumolysin plays a more important role in induction of microglia cell apoptosis.

Macrophages are known to produce various inflammatory mediators, including cytokines, in response to bacterial components such as lipopolysaccharide and peptidoglycan [32]. Oxidative stress has been implicated in the pathogenesis of a number of inflammatory diseases, including stroke and sepsis [33]. Since streptococcal H_2_O_2_ contributes to macrophage cell death, it became interesting to clarify whether H_2_O_2_ stimulates inflammatory responses such as cytokine production. The present study showed that H_2_O_2_ is not required for TNF-α production in macrophages (Figure 7). Therefore, H_2_O_2_-mediated cell death seems to be independent of the inflammatory responses of macrophages infected with oral streptococci.

Taken together, our results support the possibility that H_2_O_2_ plays a significant role in the cell death of macrophages infected with the oral mitis group of streptococci, and suggest a general role for H_2_O_2_ as a cytotoxin. The contribution of streptococcal H_2_O_2_ to the pathogenesis of infective endocarditis will be a topic of special interest for future study.

## Supporting Information

Table S1 **PCR Primers used in this study.**
(PDF)

## References

[1] Hamada S, Slade HD (1980) Biology, Immunology, and cariogenicity of *Streptococcus mutans* . Microbiol Rev 44: 331–384. 6446023 10.1128/mr.44.2.331-384.1980 PMC373181

[2] Coykendall AL (1989) Classification and identification of the viridans streptococci. Clin Microbiol Rev 2: 315–328. 2670193 10.1128/cmr.2.3.315 PMC358123

[3] Kolenbrander PE, London J (1993) Adhere today, here tomorrow: oral bacterial adherence. J Bacteriol 175: 3247–3252. 8501028 10.1128/jb.175.11.3247-3252.1993 PMC204720

[4] Nobbs AH, Lamont RJ, Jenkinson HF (2009) Streptococcus adherence and colonization. Microbiol Mol Biol Rev 73: 407–450. 19721085 10.1128/MMBR.00014-09 PMC2738137

[5] Chen L, Ge X, Dou Y, Wang X, Patel JR, et al (2011) Identification of hydrogen peroxide production-related genes in *Streptococcus sanguinis* and their functional relationship with pyruvate oxidase. Microbiol 157: 13–20. 10.1099/mic.0.039669-0 PMC3069532 20847003

[6] Zhu L, Kreth J (2012) The role of hydrogen peroxide in environmental adaptation of oral microbial communities. Oxid Med Cell Longev Article ID 717843. 10.1155/2012/717843 PMC3405655 22848782

[7] Kreth J, Zhang Y, Herzberg MC (2008) Antagonism in oral biofilms: *Streptococcus sanguinis* and *Streptococcus gordonii* interference with *Streptococcus mutans* . J Bacteriol 190: 4632–4640. 18441055 10.1128/JB.00276-08 PMC2446780

[8] Kreth J, Vu H, Zhang Y, Herzberg MC (2009) Characterization of hydrogen peroxide-induced DNA release by *Streptococcus sanguinis* and *Streptococcus gordonii* . J Bacteriol 191: 6281–6291. 19684131 10.1128/JB.00906-09 PMC2753043

[9] van der Meer JT, van Vianen W, Hu E, van Leeuwen WB, Valkenburg HA, et al (1991) Distribution, antibiotic susceptibility and tolerance of bacterial isolates in culture-positive cases of endocarditis in the Netherlands. Eur J Clin Microbiol Infect Dis 10: 728–734. 1810724 10.1007/BF01972497

[10] Douglas CW, Heath J, Hampton KK, Preston FE (1993) Identity of viridans streptococci isolated from cases of infective endocarditis. J Med Microbiol 39: 179–182. 8366515 10.1099/00222615-39-3-179

[11] Dyson C, Barnes RA, Harrison GAJ (1999) Infective endocarditis: an epidemiological review of 128 episodes. J Infect 38: 87–93. 10342647 10.1016/s0163-4453(99)90074-9

[12] Mitchell J (2011) *Streptococcus mitis*: walking the line between commensalism and pathogenesis. Mol Oral Microbiol 26: 89–98. 21375700 10.1111/j.2041-1014.2010.00601.x

[13] Health Protection Agency (2012) Pyogenic and non-pyogenic streptococcal bacteraemia (England, Wales and Northern Ireland): 2011. Health Protection Reports 6: No.46.

[14] Chiu B (1999) Multiple infections in carotid atherosclerotic plaques. Am Heart J S534–S536. 10539867 10.1016/s0002-8703(99)70294-2

[15] Koren O, Spor A, Felin J, Fak F, Stombaugh J, et al (2010) Microbes and Health Sackler Colloquium: Human oral, gut, and plaque microbiota in patients with atherosclerosis. Proc Natl Acad Sci USA 108 (Suppl 1)4592–4598. 20937873 10.1073/pnas.1011383107 PMC3063583

[16] Nakano K, Inaba H, Nomura R, Nemoto H, Takeda M, et al (2006) Detection of cariogenic *Streptococcus mutans* in extirpated heart valve and atheromatous plaque specimens. J Clin Microbiol 44: 3313–3317. 16954266 10.1128/JCM.00377-06 PMC1594668

[17] Okahashi N, Okinaga T, Sakurai A, Terao Y, Nakata M, et al (2011) *Streptococcus sanguinis* induces foam cell formation and cell death of macrophages in association with production of reactive oxygen species. FEMS Microbiol Lett 323: 164–170. 22092716 10.1111/j.1574-6968.2011.02375.x

[18] Bridge PD, Sneath PH (1982) *Streptococcus gallinarum* sp. nov. and *Streptococcus oralis* sp. nov.. Int J Syst Bacteriol 32: 410–415.

[19] Takamatsu D, Osaki M, Sekizaki T (2001) Thermosensitive suicide vectors for gene replacement in *Streptococcus suis* . Plasmid 46: 140–148. 11591139 10.1006/plas.2001.1532

[20] Okahashi N, Nakata M, Sakurai A, Terao Y, Hoshino T, et al (2010) Pili of oral *Streptococcus sanguinis* bind to fibronectin and contribute to cell adhesion. Biochem Biophys Res Commun 391: 1192–1196. 20004645 10.1016/j.bbrc.2009.12.029

[21] Sumitomo T, Nakata M, Higashino M, Jin Y, Terao Y, et al (2011) Streptolysin S contributes to group A streptococcal translation across an epithelial barrier. J Biol Chem 286: 2750–2761. 21084306 10.1074/jbc.M110.171504 PMC3024771

[22] Nakata M, Kimura KR, Sumitomo T, Wada S, Sugauchi A, et al (2011) Assembly mechanism of FCT region type 1 pili in serotype M6 *Streptococcus pyogenes* . J Biol Chem 286: 37566–37577. 21880740 10.1074/jbc.M111.239780 PMC3199502

[23] Hoshino T, Kawaguchi M, Shimizu N, Hoshino N, Ooshima T, et al (2004) PCR detection and identification of oral streptococci in saliva samples using *gtf* genes. Diagn Microbiol Infect Dis 48: 195–199. 15023429 10.1016/j.diagmicrobio.2003.10.002

[24] Takahashi N, Nyvad B (2011) The role of bacteria in the caries process: ecological perspectives. J Dent Res 90: 294–303. 20924061 10.1177/0022034510379602

[25] Spellerberg B, Cundell DR, Sandros J, Pearce BJ, Idanpaan-Heikkila, et al (1996) Pyruvate oxidase, as a determinant of virulence in *Streptococcus pneumoniae* . Mol Microbiol 19: 803–814. 8820650 10.1046/j.1365-2958.1996.425954.x

[26] Tabas I (2010) Macrophage death and defective inflammation resolution in atherosclerosis. Nat Rev Immunol 10: 36–45. 19960040 10.1038/nri2675 PMC2854623

[27] Ott M, Gogvadze V, Orrenius S, Zhivotovsky B (2007) Mitochondria, oxidative stress and cell death. Apoptosis 12: 913–922. 17453160 10.1007/s10495-007-0756-2

[28] Braun JS, Sublett JE, Freyer D, Mitchell TJ, Cleveland JL, et al (2002) Pneumococcal pneumolysin and H_2_O_2_ mediate brain cell apoptosis during meningitis. J Clin Invest 109: 19–27. 11781347 10.1172/JCI12035 PMC150815

[29] Orihuela CJ, Gao G, Francis KP, Yu J, Tuomanen EI (2004) Tissue-specific contribution of pneumococcal virulence factors to pathogenesis. J Infect Dis 190: 1661–1669. 15478073 10.1086/424596

[30] Stinson MW, Alder S, Kumar S (2003) Invasion and killing of human endothelial cells by viridans group of streptococci. Infect Immun 71: 2365–2372. 12704106 10.1128/IAI.71.5.2365-2372.2003 PMC153257

[31] Watt BE, Proudfoot AT, Vale JA (2004) Hydrogen peroxide poisoning. Toxicol Rev 23: 51–57. 15298493 10.2165/00139709-200423010-00006

[32] Ishii KJ, Koyama S, Nakagawa A, Coban C, Akira S (2008) Host innate immune receptors and beyond: making sense of microbial infection. Cell Host Microbe 3: 352–363. 18541212 10.1016/j.chom.2008.05.003

[33] Bergamini CM, Gambetti S, Dondi A, Cervellati C (2004) Oxygen, reactive oxygen species and tissue damage. Curr Pharm Des 10: 1611–1626. 15134560 10.2174/1381612043384664

