# Supplementary material for: Hydrogen Peroxide Produced by Oral Streptococci Induces Macrophage Cell Death
Source: PLoS One. 2013 May 3;8(5):e62563. doi: 10.1371/journal.pone.0062563 (PMC3643943; doi:10.1371/journal.pone.0062563)
Supplement: Table S1 — PCR Primers used in this study. (PDF) [file pone.0062563.s001.pdf]

Table S1 Okahashi et al

PCR primers used in this study

Designation	Sequence (5' to 3')	Reference
spxKO-F1	ATGGATCC-CATCTTTATTATAAGCACCCTCAC	This study
spxKO-R1	GAGAGTTATCATTATGACTCAGGATTGCAATCACGCGCAATC	
spxKO-F2	TTGCGCGTGATTGCAATCCTGAGTCATAATGATAACTCTCCTTC	This study
spxKO-R2	GCGAATTC-ATTGGTATCGAAGAGGTCATTGC	
spx-inside-F	ACCATGGAGTAGACTCAACTGG	This study
spx-inside-R	ATGGATAACACTCCATTCCTTG	
spx-outside-F	AGAGACATACATCATAACGG	This study
spx-outside-R	TCTGAGCACATTCTTAGGAG	
gtfR-F	TCCCGGTCAGCAAACTCCAGCC	Hoshino et al.
gtfR-R	GCAACCTTTGGATTTGCAAC	[23]
